# Supplementary material for: mRNAs and lncRNAs intrinsically form secondary structures with short end-to-end distances
Source: Nat Commun. 2018 Oct 18;9:4328. doi: 10.1038/s41467-018-06792-z (PMC6193969; doi:10.1038/s41467-018-06792-z)
Supplement: Supplementary file 1 — Supplementary Information [file 41467_2018_6792_MOESM1_ESM.pdf]

SUPPLEMENTARY INFORMATION FOR

mRNAs and lncRNAs intrinsically form secondary structures with short end-to-end distances

Wan-Jung C. Lai†, Mohammad Kayedkhordeh†, Erica V. Cornell, Elie Farah, Stanislav Bellaousov, Robert Rietmeijer, Enea Salsi, David H. Mathews* and Dmitri N. Ermolenko*

Department of Biochemistry & Biophysics and Center for RNA Biology, School of Medicine and Dentistry, University of Rochester, Rochester, NY 14642

Supplementary Results:

Supplementary Table 1. FRET-derived and computationally predicted end-to-end distances in various natural mRNAs, lncRNAs, and GAPDH mRNA variants.

mRNA	Species	Length (nt)	FRET derived end-to-end distance (nm)	Predicted end-to-end distance (nm)
F-Luciferase (ORF)	Firefly	1689	4.9±0.1	3.5
β-globin	Rabbit	597	4.7±0.1	4.6
Ribosomal protein L41A (RPL41A)	Yeast	321	6.7±0.2	3.5
Heat shock binding protein 1 (HSBP1)	Human	533	6.6±0.2	2.7
ATP synthase subunit F (ATP5J2)	Human	443	6.7±0.5	2.7
Macrophage migration inhibitory factor (MIF)	Human	561	4.8±0.3	3.1
Mitochondrial ribosomal protein L51 (MRPL51)	Human	690	4.3±0.4	2.6
Glyceraldehyde-3-phosphate dehydrogenase (GAPDH)	Human	1327	6.0±0.1	2.6
GAPDH 3' UTR shuffle_1	Human	1327	6.1±0.2	2.5
β-globin_polyA$_{30}$	Rabbit	627	9.9±0.4	6.5
GAPDH_polyA$_{30}$	Human	1357	11.2±0.5*	6.2
GAPDH 5' UTR(CA)$_{53}$	Human	1327	n.d.	11
GAPDH 3' UTR(CA)$_{53}$	Human	1327	n.d.	11
GAPDH 3’ UTR_NUS (genetic)	Human	1327	9.4±0.3	10.4
HOX transcript antisense RNA lncRNA (HOTAIR) lncRNA	Human	2148	6.8±0.1	4.9
Nuclear paraspeckle assembly transcript 1 lncRNA (NEAT1_S) lncRNA	Human	3734	5.7±0.2	2.1

Average FRET-derived end-to-end distances and respective SD values were determined from three to five independent mRNA and lncRNA refolding experiments performed in the presence of 1 mM $MgCl_2$ and 100 mM KCl. The computationally predicted end-to-end distances represent the mean distance in the ensemble of 1,000 structures generated by stochastic sampling with the *RNAstructure* software package for each mRNA and lncRNA. *FRET measurements of the distance between fluorophores are only reliable within the range of $2R_0$ ($2R_0 \approx 11.2$ nm for Cy3-Cy5 pair).

Supplementary Table 2. Rates of transitions between different FRET states in GAPDH mRNA.

Transition	Number of transitions	Rate, s^{-1}
0.4 → 0.6	2112	0.13 ± 0.02
0.6 → 0.4	2050	0.11 ± 0.02
0.6 → 0.8	492	0.14 ± 0.03
0.8 → 0.6	460	0.03 ± 0.01

Rates were determined from 5,114 fluctuations between different FRET states in 266 HMM-idealized FRET traces obtained for GAPDH mRNA folded in presence of 1 mM $MgCl_2$ and 100 mM KCl.

Supplementary Table 3. Primers used for cloning of mRNA-encoding sequences from HeLa cDNA.

Gene	Genebank ID	5' restriction site	3' restriction site	Sequence (5' and 3' primers)
HSBP1	NM_001537.1	HindIII	SacI	5'CCGAAGCTTTTCTAATACGACTCACTATAGGACGGAAGTGTAGGTTACGGT 3' and 5'GCCGAGCTCATGCAAGAACCGCCAAAGTTTTAG 3'
GAPDH	NM_002046	BamHI	SacI	5'CCGGGATCCTTCTAATACGACTCACTATAGGGCCTCAAGACCTTGGGCT 3' and 5'GCCGAGCTCAACTGGTTGAGCACAGGGTAC 3'
MIF	NM_002415.1	HindIII	EcoRI	5'CCGAAGCTTTTCTAATACGACTCACTATAGGACCACAGTGGTGTCCGAGAAGTC 3' and 5'GCCGAATTCAGTCTCTAAACCGTTTATTTCTCCC 3'
ATP5J2	BC003678	HindIII	SacI	5'CCGAAGCTTTTCTAATACGACTCACTATAGGGCACAGCGGACACCAG 3' and 5' GCCGAGCTCACCAGTCATGTTTTATTTGGAGGTTAATTC 3'
MRPL51	NM_016497.3	XbaI	SacI	5'CCGCCGTCTAGATTCTAATACGACTCACTATAGGGCTCGTGGCTGTTGCGGAT 3' and 5' GCCGCCGAGCTCCCAAAGAAGCCCCATTTTATTACAG 3'

Supplementary Table 4. PCR templates for cloning unstructured or randomized sequences (underlined) into the UTRs of GAPDH mRNA.

pSP64A_ GAPDH variants	5' restriction site	3' restriction site	DNA sequence of PCR templates
GAPDH_ 5' UTR $(CA)_{53}$	BamHI	Bst36I	5'GTCTGTGGATCCTTCTAATACGACTCACTATAGGCACACAC<br>ACACACACACACACACACACACACACACACACACACACACAC<br>ACACACACACACACACACACACACACACACACACACACACAC<br>ACACACACACACACACCTCAGGTCTGT 3'
GAPDH_ 3' UTR $(CA)_{53}$	KpnI	SacI	5'GGAAGAGAGGTACCCTCACTGCTGGGGAGTCCCTGCCACA<br>CTCAGTCCCCCACCACACTGCACACACACACACACACACACA<br>CACACACACACACACACACACACACACACACACACACACACA<br>CACACACACACACACACACACACACACACACACACACACACA<br>GAGCTCTCATAGCTGTTTCC 3'
GAPDH_ 3' UTR shuffle	KpnI	SacI	5'GGAAGAGAGGTACCCTCACTGCTGGGGAGTCCCTGCCACA<br>CTCAGTCCCCCACCACACTGATCCCATCTCGAAAATCACAGC<br>TCCGAAGGTTCCAGCGAGCCGCGGGCGCTGCAACCATCGTT<br>CACACTGGAATTGGCAGTGATATTACAGAACCTCGTTCCCGT<br>GGAGCTCTCATAGCTGTTTCC 3'
GAPDH_ 3' UTR Genetic	KpnI	SacI	5'GGAAGAGAGGTACCCTCACTGCTGGGGAGTCCCTGCCACA<br>CTCAGTCCCCCACCACACTGCACATTTACCCTACACATTACA<br>TTCACTTACACATTACTTACATTCCCATTCCCATACACATCCT<br>ACATACACTAACACATACATCCCACTCTAAAATACATACAAGA<br>GCTCTCATAGCTGTTTCC 3'

Supplementary Figure 1:

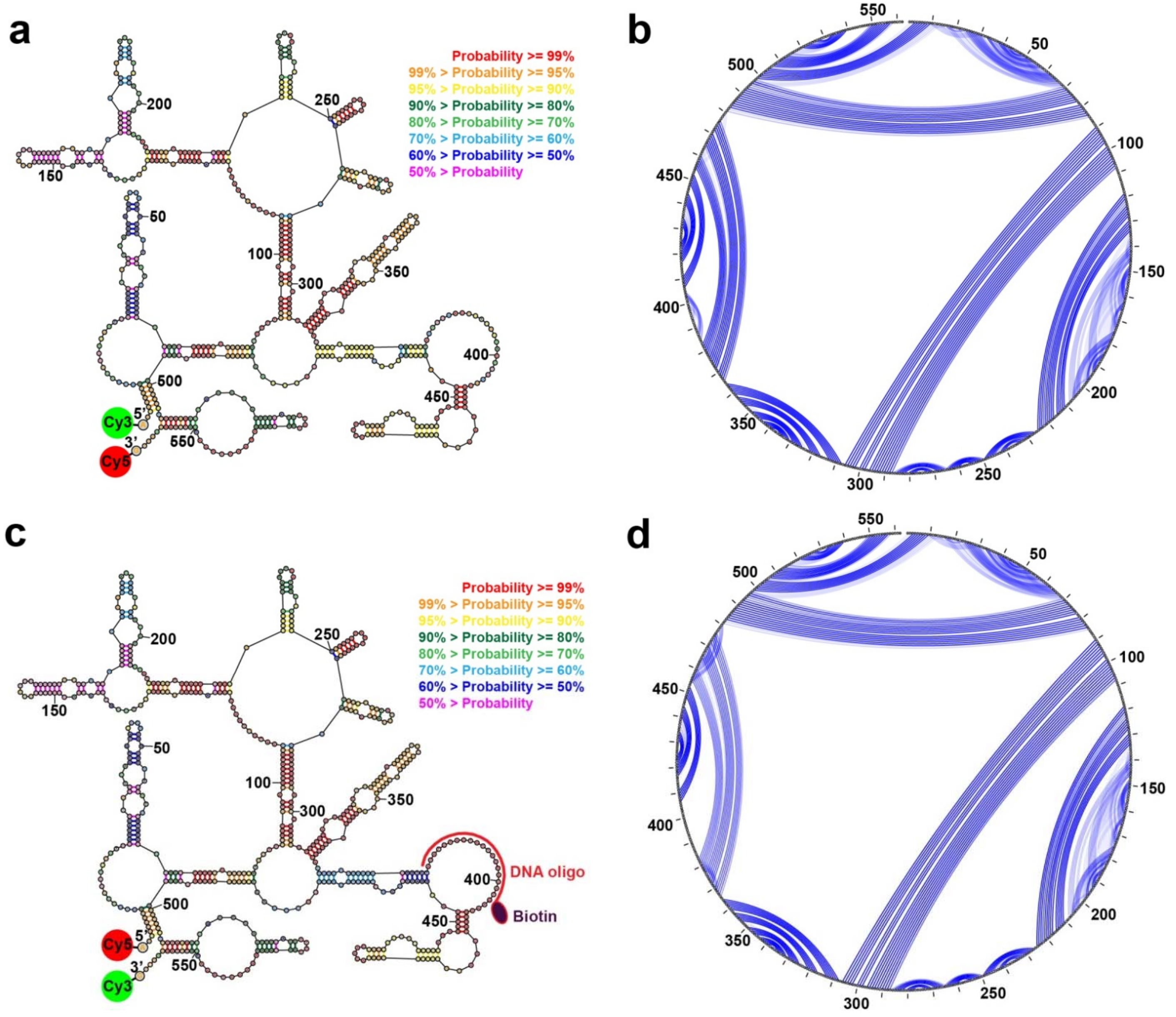

Supplementary Figure 1. Secondary structures of human *MIF* mRNA lacking poly(A) tail predicted by free energy minimization. (a) The predicted lowest free energy secondary structure of human *MIF* mRNA lacking poly(A) tail. The structure was drawn with the *StructureEditor* program in the *RNAstructure* software package *(https://rna.urmc.rochester.edu/RNAstructure.html)*. Base pair probabilities, predicted with a partition function, are indicated by the color key. In order to measure the end-to-end distance by FRET, the 5’ and 3’ ends of mRNA were conjugated with donor (green) and acceptor (red) fluorophores, respectively, as indicated. **(b)** The circle diagram depicts base pairings, which are represented by the arcs, in the secondary structure of human *MIF* mRNA lacking poly(A) tail shown in panel a. This image is produced with the *StructureEditor* program from *RNAstructure*. **(c)** Secondary structure of human *MIF* mRNA lacking poly(A) tail folded in the presence of a 20-nucleotide biotinylated DNA oligomer. The biotinylated DNA oligomer, which was designed by *OligoWalk* to have a minimal impact on the mRNA structure, was used for the immobilization of fluorescently-labeled mRNA in smFRET experiments. In order to measure the end-to-end distance by FRET, the 5’ and 3’ ends of mRNA were conjugated with acceptor (red) and donor (green) fluorophores, respectively. **(d)** The circle diagram depicts base pairings, which are represented by the arcs, in the structure shown in panel **c**.

Supplementary Figure 2:

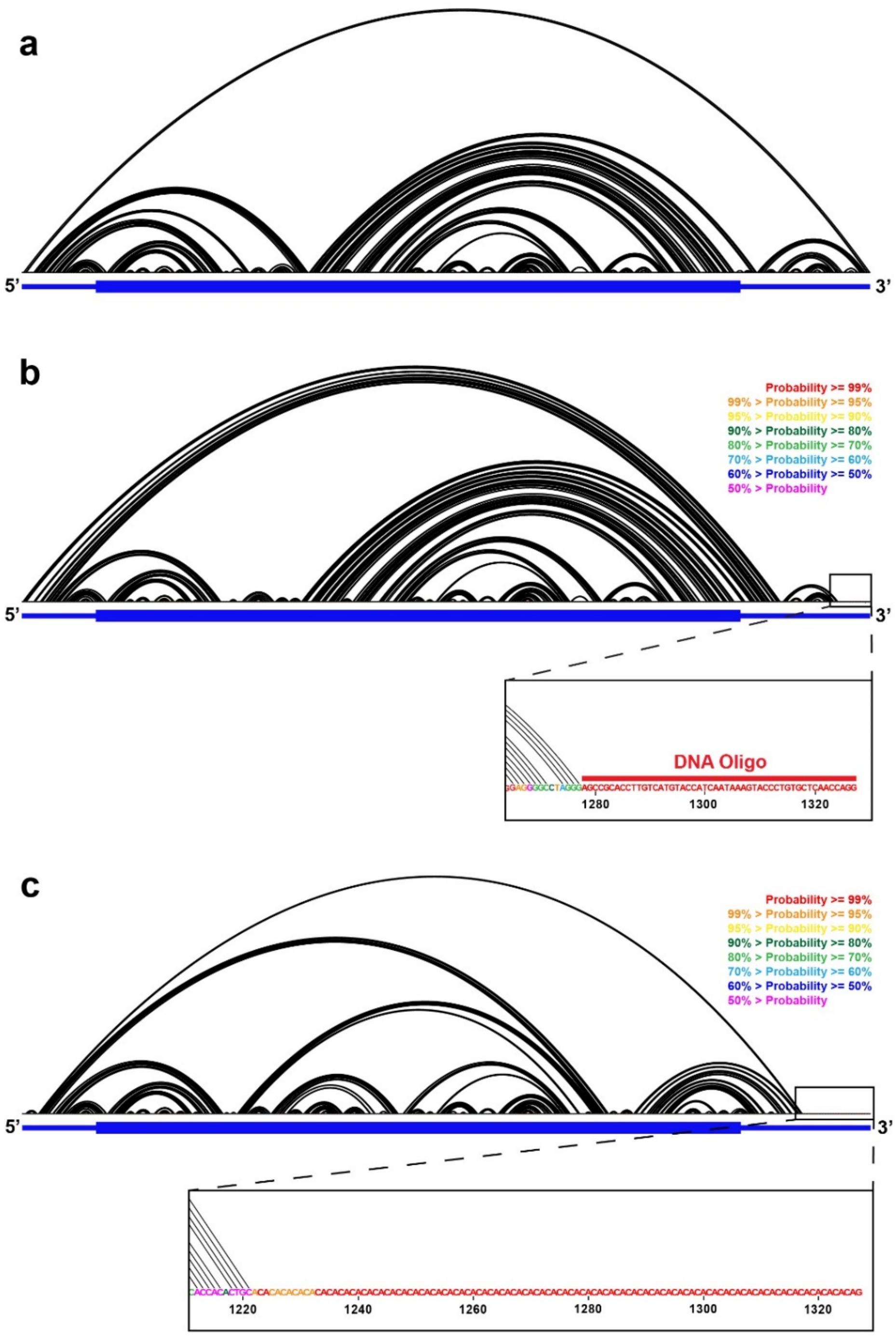

Supplementary Figure 2: Secondary structure linear diagrams for GAPDH variants. Predicted minimum free energy (MFE) secondary structures of GAPDH sequences are shown as linear diagrams: a) wild-type GAPDH; b) wild-type GAPDH with a complementary 50 nucleotide DNA-oligo annealed to the 3' end of the 3' UTR; c) GAPDH mRNA variant containing 53 CA repeats introduced into the 3' UTR. Each arc represents a base pair between two nucleotides. ORF is indicated by thick line. For panels b and c, the 3' end of the sequences are enlarged to show the nucleotides. These are color-coded (according to the legends) to show the probabilities of base pairs for paired nucleotides and the probabilities of being unpaired for unpaired nucleotides. The probabilities are predicted with a partition function.

Supplementary Figure 3:

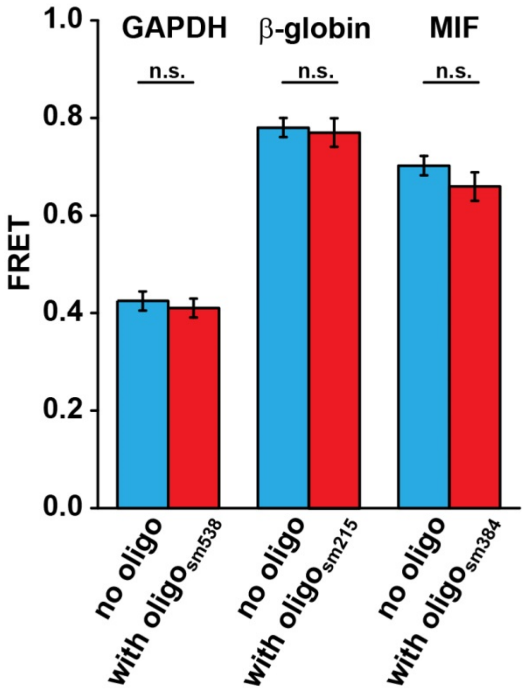

Supplementary Figure 3. Annealing of biotin-labeled DNA oligomers does not affect end-to-end distance in GAPDH, β-globin and MIF mRNA. Biotinylated DNA oligonucleotides (sm538 for GAPDH mRNA, sm215 for β-globin mRNA, and sm384 for MIF mRNA), which were predicted to have a minimal effect on the overall secondary structure and end-to-end distance, were used to tether the mRNAs to the microscope slide in smFRET experiments shown in **Fig. 2**. FRET values measured in mRNAs folded in the presence (red) or in the absence (blue) of biotinylated DNA oligomers were not statistically significant (n.s.), as determined by the Student t-test (with α of 0.05). Each FRET value represents the mean ± SD of three independent experiments.

Supplementary Figure 4:

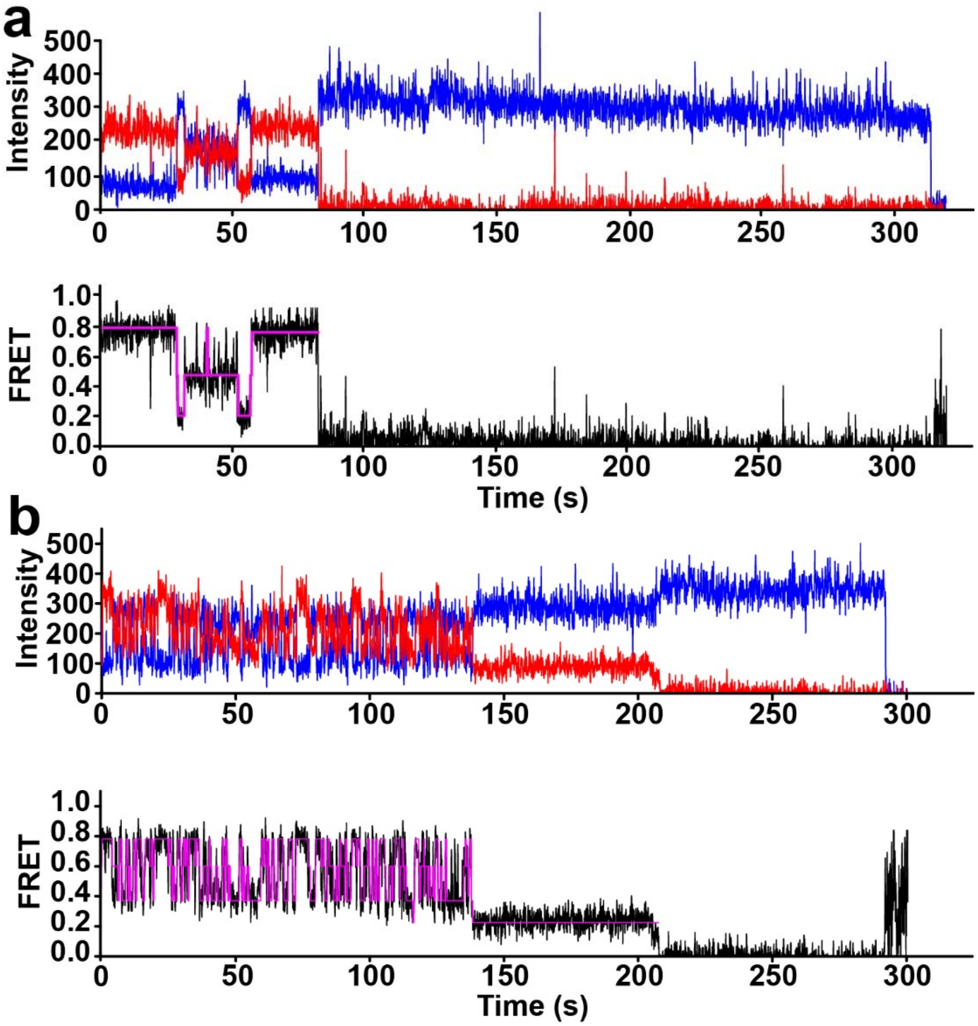

Supplementary Figure 4. smFRET traces for GAPDH mRNA showing fluctuations between 0.2, 0.4 and 0.8 (panel a) or 0.2, 0.4, 0.6 and 0.8 FRET states (panel b). Observed intensities of donor and acceptor fluorescence and the calculated apparent FRET efficiency are shown in blue, red and black, respectively. The Hidden Markov Model fit is shown in magenta.

Supplementary Figure 5:

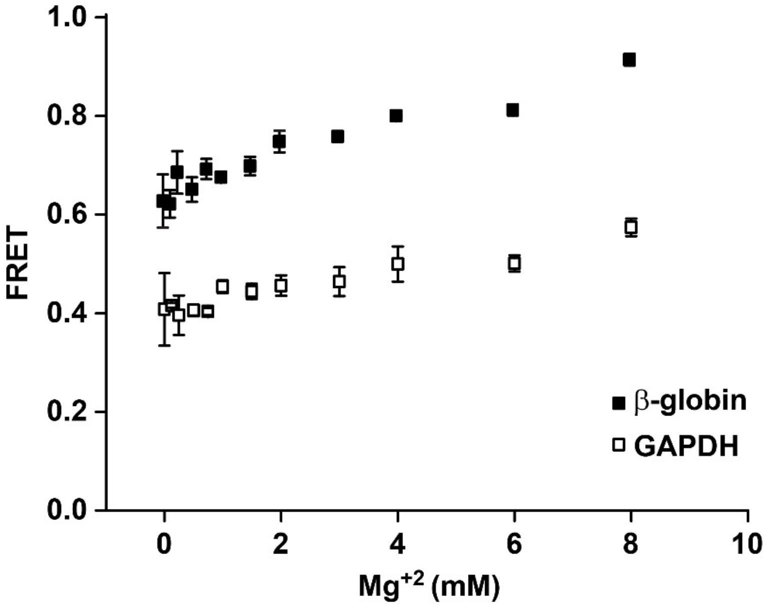

Supplementary Figure 5. Efficiency of energy transfer between fluorophores attached to mRNA ends as a function of $MgCl_2$ concentration. GAPDH (open squares) and β-globin (filled squares) mRNAs were folded in the presence of different $MgCl_2$ concentrations ranging from 0 to 8 mM. Error bars show standard deviations calculated from three to five independent experiments.

Supplementary Figure 6:

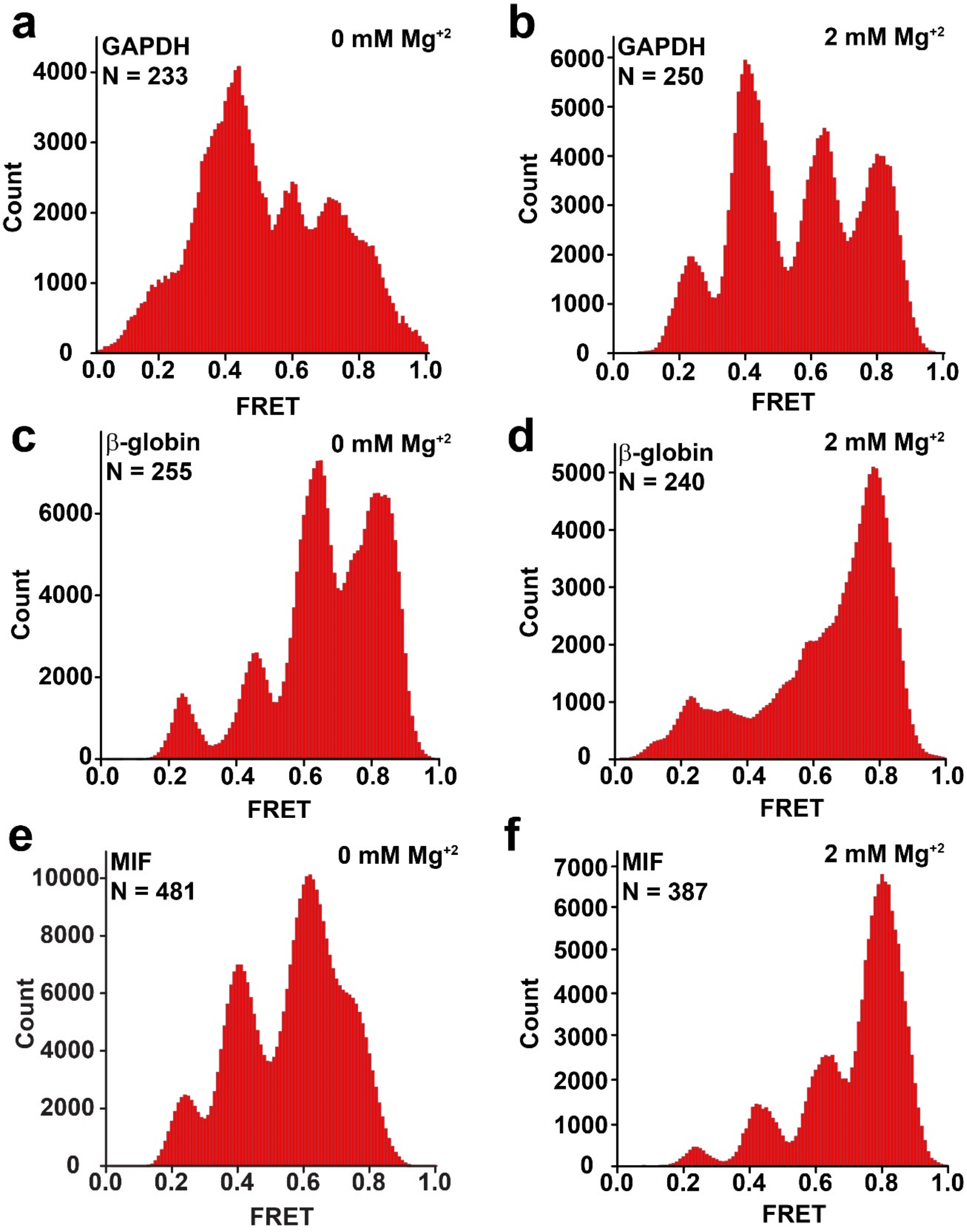

Supplementary Figure 6. FRET distribution histograms for GAPDH (a-b), β-globin (c-d) and MIF (e-f) mRNAs folded and imaged in the absence of $MgCl_2$ (a, c, e) or in the presence of 2 mM $MgCl_2$ (b, d, f). N is the number of traces used to assemble each histogram.

Supplementary Figure 7:

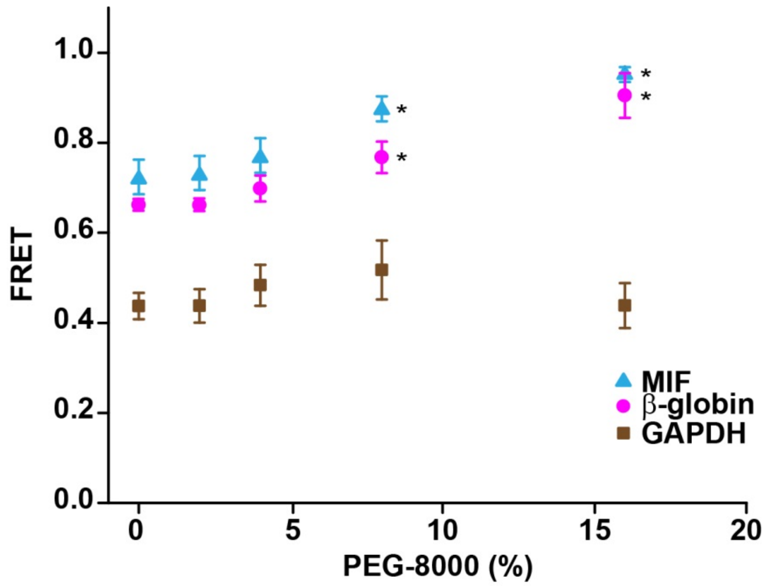

Supplementary Figure 7. Efficiency of energy transfer between fluorophores attached to mRNA ends as a function of PEG-8000 concentration. GAPDH (brown), β-globin (magenta), and MIF (blue) mRNAs were folded in the presence of 100 mM KCl, 1 mM $MgCl_2$ and different PEG-8000 concentrations ranging from 0 to 16% (wt/vol). Error bars show standard deviations calculated from three independent experiments. A star indicates that FRET values are different from those measured in the absence of PEG-8000, as p-values determined by Student's t-test were below 0.05.

Supplementary Figure 8:

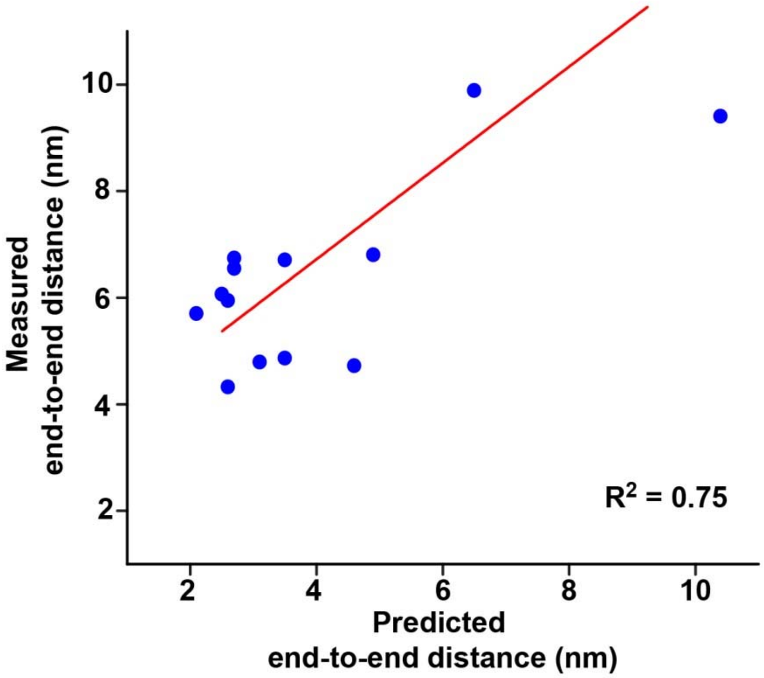

Supplementary Figure 8. Correlation between computationally predicted (x-axis) and experimentally determined (y-axis) end-to-end distances. End-to-end distance was predicted and measured by FRET in yeast RPL41A, firefly luciferase, rabbit β-globin, human ATP5J2, HSBP1, MIF, MRPL51, GAPDH mRNAs (wild-type, GAPDH 3' UTR shuffle_1 and GAPDH 3' UTR_NUS), HOTAIR, and NEAT1_S lncRNAs, all of which lacked poly(A) tail. In addition, end-to-end distance was predicted and measured by FRET in rabbit β-globin containing a 30 nt-long poly(A) tail.

Supplementary Figure 9:

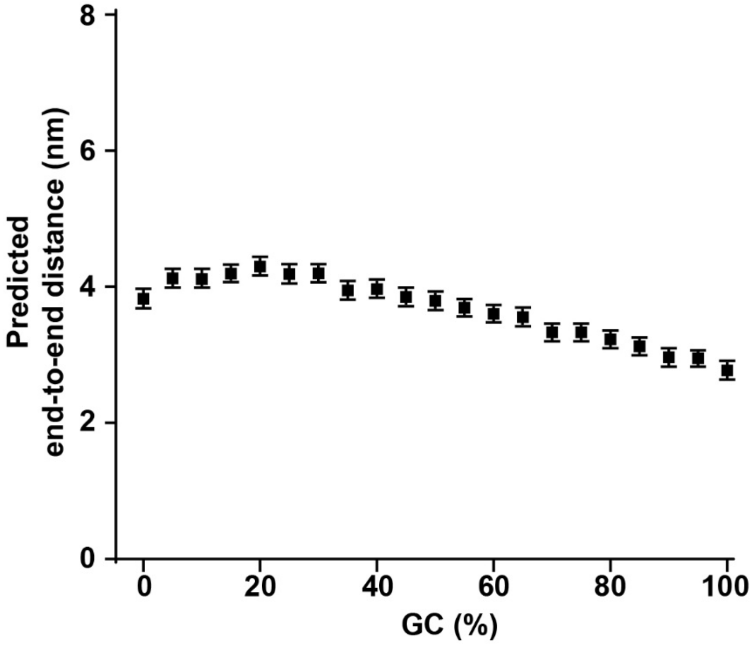

Supplementary Figure 9. Variation of GC content of a 1327 nucleotide-long RNA sequences does not substantially alter average end-to-end distances. Average end-to-end distances of GAPDH sequences are shown versus their GC content. Each data point is calculated by averaging over 100 sequences, and the standard error of the mean is plotted.

Supplementary Figure 10:

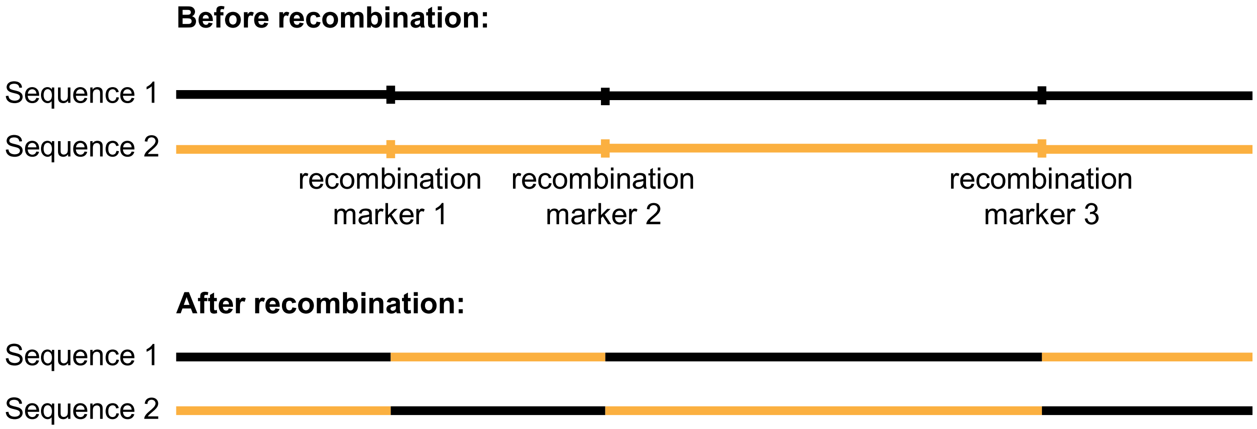

Supplementary Figure 10. Genetic algorithm crossover. In crossover, two new sequences are generated from two sequences in the population. The black and orange colors indicate the sequence origin and final sequences. Recombination markers are chosen at random with frequency of 0.03 per nucleotide.

Supplementary Methods:

Experimental procedures

Cloning of RNA-encoding sequences

Human cDNA was used to clone mRNA-encoding sequences. To prepare human cDNA, total RNA was first extracted from HeLa cells using TRIzol® reagent (Invitrogen Life Technologies) according to the manufacturer's protocol. Genomic DNA was removed from the sample by DNase treatment (NEB). RT-PCR was performed to synthesize cDNA using 5 µg of RNA, SuperScript III Reverse Transcriptase (Invitrogen Life Technologies) and oligo dT_{23} (Sigma), following the manufacturers' protocols. The target genes were amplified by PCR using Q5 DNA polymerase (NEB) and 5' and 3' primers listed in **Suppl. Table 3**. All 5' primers contain a restriction site for cloning, a T7 promoter sequence (5' TTCTAATACGACTCACTATAGG 3'), and the sequence complementary to the 5' end of the 5' UTR. All 3' primers contain the sequence complementary to the 3' end of the 3' UTR and a restriction site for cloning.

Primers were designed based on general guidelines using the IDT *OligoAnalyzer* tool. The optimal annealing temperature for each primer pair was determined using the *NEB Tm Calculator* to set the PCR conditions. A 30 second annealing step 3°C above the Tm was used after the initial denaturation step of 30 seconds at 95°C. The extension temperature was set to 72°C for 1 min per kb. The PCR products were cloned into polylinker sites of the pSP64A vector (Promega), where the 3' cloning site has a stretch of 30 dA:dT residues inserted between the SacI and EcoRI restriction sites. Linearization of the recombinant plasmid with EcoRI was performed for the use of run-off *in vitro* transcription by T7 RNA polymerase to prepare RNA with a synthetic 30-nt poly(A) tail.

The rabbit β-globin gene was amplified directly from rabbit globin mRNA purchased from Sigma using the forward (5' TCGAGTAAGCTTACACTTGCTTTTGACACAACTGTG 3') and reverse (5' CATACAGAGCTCGCAATGAAAATAAATTTCCTTTATTAGC 3') primers and cloned into the pSP64A vector using HindIII and SacI. Because an EcoRI restriction site is present in the sequence encoding β-globin mRNA, the second EcoRI site positioned downstream of the poly(A) track was mutated into an AgeI restriction site via the QuikChange Site-Directed Mutagenesis System (Stratagene) using internal mutagenic primers by the AgeI

site (5' AAAAAAAAAACCGGTTTCTGCAGATATCCATCACACTGGCGGCCG 3' and 5' CGGCCGCCAGTGTGATGGATATCTGCAGAAACCGGTTTTTTTTTT 3').

The plasmid containing the HOTAIR lncRNA sequence (NR_047517)[1] was purchased from Addgene (LZRS-HOTAIR, #26110). The HOTAIR sequence was cloned into the pSP64A vector after PCR amplification with a forward primer (5' CCCGGGTTCTAATACGACTCACTATAGGGACTCGCCTGTGCTCTGGAGCTTGAT 3'), which contained the T7 promoter sequence and XmaI restriction site, and the reverse primer (5' GAGCTCGAAAATGCATCCAGATATTAATATATCTAC 3'), which contained the SacI restriction site.

The pcDNA3-FLUC plasmid containing the coding sequence of Firefly luciferase was a gift from Prof. Nahum Sonenberg[2]. The plasmid encoding yeast RPL41A was a gift from Prof. Jon Lorsch[3]. The plasmid containing the human NEAT1_S sequence (NR_028272.1)[4] was purchased from Addgene (pCRII_TOPO_hNEAT1, #61518). All plasmids were propagated in *E. coli* strain TOP10F' competent cells.

Construction of GAPDH variants

To obtain the construct containing 53 CA repeats in the 5' UTR of the GAPDH mRNA [abbreviated: GAPDH_5' UTR $(CA)_{53}$], the T7 promoter and 106 bp in the 5' end of the 5' UTR were excised from the GAPDH-encoding plasmid via digestion with BamHI and Bst36I and replaced with a fragment listed in **Suppl. Table 4** (GAPDH_5' UTR $(CA)_{53}$), which contained the T7 promoter sequence and 53 CA repeats. The fragment was PCR amplified using forward (5' GTCTGTGGATCCTTCTAATACG 3') and reverse (5'ACAGACCTGAGGTGTGTG 3') primers and digested using BamHI and Bst36I. The construct containing 53 CA repeats in the GAPDH 3' UTR [GAPDH_3' UTR $(CA)_{53}$] was generated by replacing the 152 bp KpnI-SacI sequence in the 3' end of the 3' UTR with a fragment containing the original 46 bp sequence downstream of the KpnI site and 53 CA repeats (indicated in **Suppl. Table 4**). The latter fragment was PCR amplified using forward (5' AAGAGAGGTACCCTCACTGCT 3') and reverse (5'GGAAACAGCTATGAGAGCTC 3') primers and digested using KpnI and SacI. The GAPDH constructs containing randomized or genetic sequences [GAPDH_3' UTR shuffle; GAPDH_3' UTR Genetic] in the GAPDH 3' UTR were generated as described above by replacing the 152 bp KpnI-SacI sequence at the 3' UTR of GAPDH with the fragments indicated in **Suppl. Table 4**.

Computational procedures

Variation of GC content

To vary GC content (**Suppl. Fig. 9**), we generated sequences at random with length 1327 nt (equal in length to GAPDH). The G+C content was varied from 0% to 100%. The ratios A/U and G/C were fixed to the ratios observed in GAPDH. To generate 100 sequences for each G+C content, the original random sequence for each G+C content was shuffled.

Sequence complexity

Sequence complexity is a measure of diversity for the nucleotide content of a sequence. In this work, we use *Linguistic complexity* as introduced by Trifonov[5], and calculated using the algorithm from Gabrielian et al[6]. The complexity is the product of vocabulary size across k-mers:

$$C = \prod_{k=1}^{w} U_k$$

where the vocabulary size, *U*, is the fraction of possible sequences observed for that k-mer. The number of possible sequences for a k-mer is the minimum of 4^k or N-k+1, where N is the sequence length. For example, $k = 3$ has a possible sequence space of 4^3 for sequences of 64 or more nucleotides, and U_3 is the fraction of these 3-mer sequences observed across the sequence. The maximum k-mer size, *w*, is a function of length. Here we used $w = 5$ for the 106 nucleotide region of GAPDH mRNA and $w = 7$ for the full length GAPDH mRNA, following Gabrielian et al[6].

Genetic algorithm

We developed a genetic algorithm program to optimize features in a given RNA sequence. In this work, our goal was to evolve sequences to increase the end-to-end distance of the input sequences.

The genetic algorithm is an iterative process inspired by evolution in which an initial population is evolved to optimize features represented in the objective function[7]. A population of 10 sequences was used in this work, and these ten sequences were initialized uniformly as the starting sequence. In each iteration, sequences in the population are either mutated or new sequences are generated by recombining two sequences (called crossover) to generate 10 new sequences (**Suppl. Fig. 10**). The optimal 10 sequences (from the set of 10 at the start of the iteration and the 10 new sequences) are kept for subsequent iterations, where optimality is

defined as maximizing the value of the objective function. In the mutation steps, each of the ten sequences was mutated independently. Sweeping along the portion of the sequence that is being evolved, there is a probability of 0.03 that a nucleotide will mutate to equal probability of A, C, G, or U. In our algorithm, crossover occurred every 6 steps. For crossover, 5 pairs of sequences are selected at random without replacement from the population of 10 sequences. For each sequence pair, the algorithm scans through the portion of the sequence that is being evolved and each nucleotide position has a probability of 0.03 to be selected as a recombination marker; therefore, on average, the number of recombination markers is $0.03\times N$. Then, the pair of sequences is recombined by the exchange of homologous segments to make two new sequences. The generation of the two sequences by crossover from the sequence pair is illustrated by the schematics shown below.

We used two objective functions in this work. In calculations shown in **Fig. 5**, the objective function was the mean probability of each nucleotide being unpaired as determined with a partition function calculation[8]. The mean is taken across only nucleotides that are in the region of the sequence being evolved. In calculations shown in **Fig. 6**, we summed the mean probability of each nucleotide being unpaired and the sequence complexity.

Supplementary References:

1. Gupta, R.A. et al. Long non-coding RNA HOTAIR reprograms chromatin state to promote cancer metastasis. *Nature* **464**, 1071-6 (2010).
2. Poulin, F., Gingras, A.C., Olsen, H., Chevalier, S. & Sonenberg, N. 4E-BP3, a new member of the eukaryotic initiation factor 4E-binding protein family. *J Biol Chem* **273**, 14002-7 (1998).
3. Mitchell, S.F., Walker, S.E., Algire, M.A., Park, E.H., Hinnebusch, A.G. & Lorsch, J.R. The 5'-7-methylguanosine cap on eukaryotic mRNAs serves both to stimulate canonical translation initiation and to block an alternative pathway. *Mol Cell* **39**, 950-62 (2010).
4. Clemson, C.M., Hutchinson, J.N., Sara, S.A., Ensminger, A.W., Fox, A.H., Chess, A. & Lawrence, J.B. An architectural role for a nuclear noncoding RNA: NEAT1 RNA is essential for the structure of paraspeckles. *Mol Cell* **33**, 717-26 (2009).
5. Trifonov, E.N. Making sense of the human genome. in *Structure and methods*, Vol. 1 (eds. Sarma, R.H. & Sarma, M.H.) 69-77 (Adenine Press, 1990).
6. Gabrielian, A. & Bolshoy, A. Sequence complexity and DNA curvature. *Comput Chem* **23**, 263-74 (1999).
7. Forrest, S. Genetic algorithms: principles of natural selection applied to computation. *Science* **261**, 872-8 (1993).
8. Mathews, D.H. Using an RNA secondary structure partition function to determine confidence in base pairs predicted by free energy minimization. *RNA* **10**, 1178-90 (2004).
